# Scorpion venom peptide HsTx2 suppressed PTZ-induced seizures in mice via the circ_0001293/miR-8114/TGF-β2 axis

**DOI:** 10.1186/s12974-022-02647-z

**Published:** 2022-12-01

**Authors:** Yan Hu, Buliang Meng, Saige Yin, Meifeng Yang, Yilin Li, Naixin Liu, Shanshan Li, Yixiang Liu, Dandan Sun, Siyu Wang, Yinglei Wang, Zhe Fu, Yutong Wu, Ailan Pang, Jun Sun, Ying Wang, Xinwang Yang

**Affiliations:** 1 Department of Anatomy and Histology and Embryology, Faculty of Basic Medical Science, Kunming Medical University, Kunming, 650500 Yunnan China; 2 Key Laboratory of Chemistry in Ethnic Medicine Resource, State Ethnic Affairs Commission & Ministry of Education, School of Ethno-Medicine and Ethno-Pharmacy, Yunnan Minzu University, Kunming, 650504 Yunnan China; 3 Department of Gynecology, Third Affiliated Hospital of Kunming Medical University, Kunming, 650118 Yunnan China; 4 Department of Neurology, First Affiliated Hospital of Kunming Medical University, Kunming, 650031 Yunnan China

**Keywords:** Epilepsy, Peptide, circ_0001293/miR-8114/TGF-β2, NF-κB signaling pathway, MAPK signaling pathway

## Abstract

**Background:**

Due to the complexity of the mechanisms involved in epileptogenesis, the available antiseizure drugs (ASDs) do not meet clinical needs; hence, both the discovery of new ASDs and the elucidation of novel molecular mechanisms are very important.

**Methods:**

BALB/c mice were utilized to establish an epilepsy model induced by pentylenetetrazol (PTZ) administration. The peptide HsTx2 was administered for treatment. Primary astrocyte culture, immunofluorescence staining, RNA sequencing, identification and quantification of mouse circRNAs, cell transfection, bioinformatics and luciferase reporter analyses, enzyme-linked immunosorbent assay, RNA extraction and reverse transcription–quantitative PCR, Western blot and cell viability assays were used to explore the potential mechanism of HsTx2 via the circ_0001293/miR-8114/TGF-β2 axis.

**Results:**

The scorpion venom peptide HsTx2 showed an anti-epilepsy effect, reduced the inflammatory response, and improved the circular RNA circ_0001293 expression decrease caused by PTZ in the mouse brain. Mechanistically, in astrocytes, circ_0001293 acted as a sponge of endogenous microRNA-8114 (miR-8114), which targets transforming growth factor-beta 2 (TGF-β2). The knockdown of circ_0001293, overexpression of miR-8114, and downregulation of TGF-β2 all reversed the anti-inflammatory effects and the influence of HsTx2 on the MAPK and NF-κB signaling pathways in astrocytes. Moreover, both circ_0001293 knockdown and miR-8114 overexpression reversed the beneficial effects of HsTx2 on inflammation, epilepsy progression, and the MAPK and NF-κB signaling pathways in vivo.

**Conclusions:**

HsTx2 suppressed PTZ-induced epilepsy by ameliorating inflammation in astrocytes via the circ_0001293/miR-8114/TGF-β2 axis. Our results emphasized that the use of exogenous peptide molecular probes as a novel type of ASD, as well as to explore the novel endogenous noncoding RNA-mediated mechanisms of epilepsy, might be a promising research area.

**Graphical Abstract:**

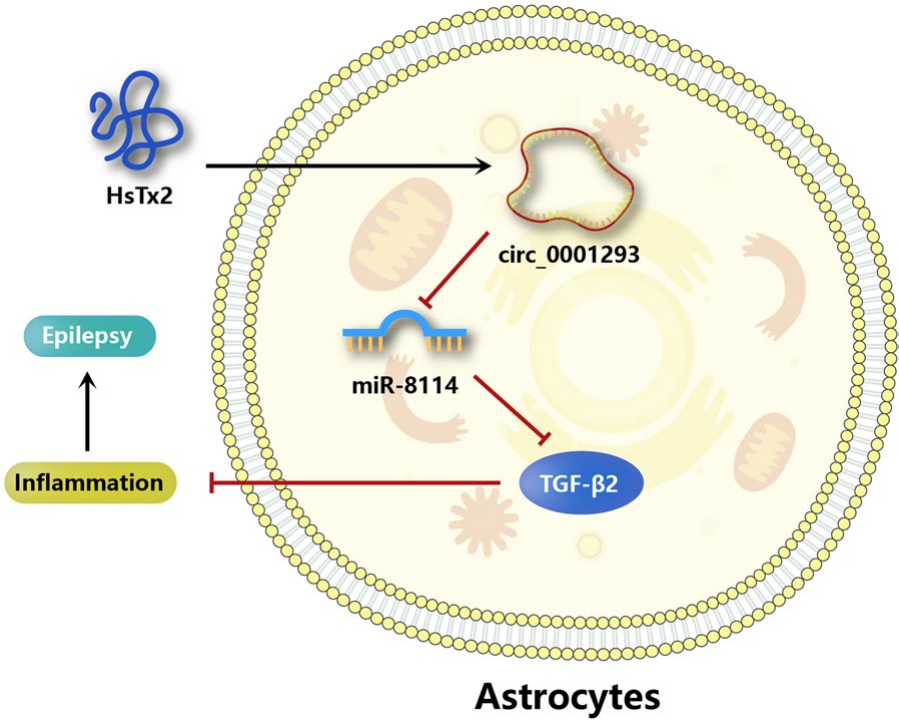

## Introduction

Epilepsy is a common, serious neurological condition causing recurrent unprovoked seizures. It is the fourth most common neurological disorder and affects ~ 65 million people worldwide [1]. The current antiseizure drugs (ASDs) primarily target voltage-gated ion channels to directly reduce neuronal excitability or synaptic transmission [2]. Despite the existence of multiple antiepileptic drugs, many patients are diagnosed with intractable epilepsy [3]. Therefore, the discovery of new ASDs may provide new hope for the treatment of epilepsy.

Epileptogenesis is associated with an increased, strong, and persistent inflammatory state in the microenvironment of neural tissue [4]. Neuronal excitability and inflammation are the main mechanisms by which abnormal glial cells promote epilepsy. Thus, activation of microglia and astrocytes upregulates various prototypical inflammatory cytokines, including interleukin-1β (IL-1β), interleukin-6 (IL-6), and tumor necrosis factor-α (TNF-α) [5]. These inflammatory cytokines denature and neurotoxicize neurons in brain tissue, resulting in proinflammatory effects. Therefore, inhibition of inflammatory factor levels, such as application of an IL-1 receptor antagonist (IL-1ra) or increased anti-inflammatory factor levels, such as IL-10, has been reported to inhibit the inflammatory response in the nervous system and play an anticonvulsant role [6]. Astrocytes are the major glial cell type in the central nervous system (CNS). Reactive astrocytes release proinflammatory cytokines and recruit additional inflammatory cells by secreting C–C motif chemokine ligands 2, 3, and 5 [7], suggesting that astrocytes play a major role in regulating the inflammatory response in several human CNS diseases, including epilepsy [8, 9]. Notably, astrocytes produce transforming growth factor beta (TGF-β) [10]. TGF-β, a pleiotropic cytokine family member, has been reported to be involved in the pathogenesis of many diseases, such as stroke, tumors, Alzheimer's disease, and epilepsy. In an epileptic model with an open blood‒brain barrier, the TGF-β signaling pathway is activated, and blocking the TGF-β signaling pathway can reduce the occurrence of cerebral inflammation and spontaneous seizures [11]. In addition, the dysfunction of astrocytes has been reported to be related to seizures. The transformation of astrocytes is considered to begin in the latency of epilepsy and is regulated by the TGF-β signaling pathway [12]. Furthermore, low levels of TGF-β are reported in the brains of epileptic patients, while the activation of astrocytes increases the level of TGF-β. Therefore, TGF-β is considered to be a potential therapeutic target for epilepsy [13]. During epileptic seizures, a cerebral inflammatory reaction will also occur. At this time, a variety of inflammation-related signaling pathways, such as MAPK and NF-κB, are involved [14] can be activated. Therefore, inhibiting the activation of these signaling pathways may inhibit the epileptic process as well. In previous research, we found that HsTx2 had a neuroprotective effect by activating the MAPK signaling pathway in cerebral ischemia rats (upregulation of ERK1/2 and p38 phosphorylation levels and inhibition of JNK phosphorylation levels) [15]. This finding indicated that HsTx2 might reduce abnormal inflammatory reactions in the brain by inhibiting the MAPK signaling pathway, which was also indicated in this research.

Since 2000, approximately 30 innovative peptide drugs have been approved and marketed, with several achieving great success, such as abaloparatide, semaglutide, and plecanatide [16, 17]. In recent years, numerous toxins purified from the venom of arthropods have been shown to possess antiseizure activity [18]. For example, BmKIT2, a sodium channel-specific neurotoxin from the Asian scorpion *Buthus martensi* Karsch, inhibits the widespread seizures induced by pentylenetetrazol (PTZ) and pilocarpine-induced status epilepticus in rats [19]. Parawixin2, a compound isolated from *Parawixia bistriata* spider venom, exerts promising neuroprotective and antiseizure effects [20, 21]. Ppnp7, extracted from the venom of the social wasp *Polybia paulista*, exerted a noticeable antiseizure effect on rats and mice [22]. These neurotoxins act on ion channels or receptors expressed in the mammalian nervous system with marked specificity [18]. In our previous study, a novel peptide named HsTx2 (AGKKERAGSRRTKIVMLKCIREHGH, 2 861.855 Da), which is derived from the scorpion *Heterometrus spinifer,* was identified, and HsTx2 exerted obvious neuroprotective effects on rats with ischemic stroke via the MAPK signaling pathway [15]. Considering the critical roles of the MAPK signaling pathway in the process of epilepsy, HsTx2 may be a new potential ASD.

MicroRNAs are powerful modulators of post-transcriptional gene expression that are dysregulated during epileptogenesis [23]. They are important regulators of gene expression and modulators of inflammatory responses. MiR-146a, miR-155 and miR-21 have been shown to be critical for the immune response and Toll-like receptor (TLR) modulation. Furthermore, many miRNAs exist in glial-derived cells, including astrocytes, and likely play a role in neuroinflammatory signaling pathways [24].

Circular RNAs (circRNAs) are a subclass of endogenous noncoding RNAs that form covalently closed loops without 5′ to 3′ polarity or polyadenylated tails [25, 26]. Recently, circRNAs have been reported to be involved in various human diseases, including cancer and neurological, muscular and cardiovascular disorders [27, 28]. Mechanistically, some circRNAs exert important biological functions by acting as microRNAs or protein inhibitors ('sponges') by regulating protein function or by being translated themselves [28]. In epilepsy, circRNAs have been reported to be important endogenous genes that regulate epilepsy progression [29–31]. Despite improvements in our understanding of circRNAs, the potential correlation between circRNAs and epilepsy progression has not been fully elucidated.

In this study, we first explored the anti-seizure effects of HsTx2 and the underlying mechanisms and found that peptide HsTx2 had no effect on potassium and calcium channels (data not shown); thus, the neuroprotective mechanism of HsTx2 was not clear. A cell membrane penetration experiment showed that the peptide HsTx2 may cross the membrane of astrocytes directly (data not shown). We also analyzed the expression profiles of circRNAs in hippocampal tissues from epileptic mice and identified a circular RNA termed circ_0001293. After detecting inflammatory factors in the brain tissue of PTZ model mice, knockdown of circ_0001293 and overexpression of miR-8114 in astrocytes, HsTx2 was finally considered to alter the inflammatory response in seizures by regulating the circ_0001293/miR-8114/TGF-β2 axis. According to our results, HsTx2 has the potential to serve as a new potential ASD.

## Materials and methods

### HsTx2 synthesis

The peptide HsTx2 (purity > 95%) and the random scrambled peptide (AGERSRKILKREHGHICMVTRGAKK) (purity > 95%) were commercially synthesized and provided by Wuhan Bioyeargene Biotechnology Co., Ltd. (China).

### Animals

BALB/c mice (weight 20–22 g; 8 weeks) were purchased from Hunan SJA Laboratory Animal Co., Ltd. (Certificate no. 43004700043639, China) and housed under laboratory conditions (relative humidity of 45–55%, 12-h light/dark cycle, freely available food and water) at room temperature of 20–23 °C. All experimental protocols were approved by the Animal Experimental Ethical Inspection of Kunming Medical University (KMMU2020076). The epilepsy mouse model was induced by administering PTZ (Sigma–Aldrich, St. Louis, MO, USA) [32]. Briefly, mice were intraperitoneally injected with PTZ (35 mg/kg) once every other day for a total of 10 injections (from day 1 to day 20). After each injection, all animals were immediately observed for 30 min. Mice presenting at least three consecutive seizures with a score of 4 or 5 were considered fully kindled. Dosing was stopped for 1 week, and the model mice were used for experiments. The mice were randomly divided into the following groups. Each group was first given an intraperitoneal injection of pretreatment. PTZ was intraperitoneally injected after 30 min of pretreatment, and behavior observation and/or EEG monitoring were started immediately after PTZ was administered. The mice were randomly divided into the following groups: (1) NC group: mice receiving 0.9% (W/V) NaCl (intraperitoneally, 0.4 mL); (2) PTZ group: mice receiving PTZ (as described above); (3) ethosuximide group (positive control): mice receiving ethosuximide and PTZ; (4) PTZ + HsTx2 group: mice receiving HsTx2 (intraperitoneally, 0.5 and 1 nmol/kg) and PTZ; (5) PTZ + random scrambled peptide group: mice receiving random scrambled peptide (intraperitoneally, 1 nmol/kg) and PTZ; (6) PTZ + HsTx2 + si-circ group: mice receiving HsTx2, si_0001293 (knockdown of circ_0001293 induced by the intrahippocampal injection of a specific siRNA) and PTZ; and (7) PTZ + HsTx2 + si-circ + miR mimic group: mice receiving HsTx2, si_0001293, agomiR-8114 mimic (overexpression of miR-8114 induced by an intrahippocampal injection of agomiR-8114 mimic) and PTZ.

Thirty minutes after each behavioral observation experiment, the mice were anesthetized with 4% chloral hydrate solution and then intracardially perfused with phosphate-buffered saline for 3 min, and the hippocampal tissue was collected. Protein samples were used immediately for biochemical assays or stored in a − 80 °C freezer for total RNA extraction and western blot testing.

### Electrophysiological and behavioral observations

Behavioral seizure scoring and electrophysiological (EEG) evaluations were performed at the last PTZ administration. Two unipolar scalp electrodes were placed on the bilateral temporal skin of the mice. Then, the mice were allowed to move freely in transparent cages. The baseline EEG was recorded for approximately 15 min before the injection of PTZ or saline, and then the EEG was recorded for at least 30 min using the numerical acquisition system (BIOPAC System Inc., Goleta, CA, USA, Model MP150). An electrophysiological seizure was defined as a seizure with a high frequency (> 5 Hz) and high amplitude (> 2 times the baseline) that lasted for more than 5 s. The seizure intensity was assessed based on the Racine scale: stage 0, no response; stage 1, mouth and facial movements; stage 2, head nodding; stage 3, forelimb clonus; stage 4, rearing; stage 5, rearing and falling; stage 6, death [33]. The behavioral data captured by the synchronized video recording system were used to confirm EEG seizure activity.

### Primary astrocyte culture

Primary astrocyte cultures were prepared from the hippocampal tissue of postnatal BALB/c mice on day 1 as previously described [34]. Cell culture reagents for astrocytes were purchased from Thermo Fisher Scientific (Waltham, MA). Hippocampal tissues were isolated and trypsinized. Cells were centrifuged, and the supernatants were removed. The pellets were suspended and cultured. The cultures were further purified by shaking after reaching 90% confluence. The culture medium was changed twice a week. Each time, the culture was pipetted up and down gently to remove loosely attached oligodendrocytes, microglia, and neurons. Purified ACSC2 + cells were then obtained by staining cultures with an anti-ACSC2-APC antibody (Miltenyi Biotec, Germany) and subjecting them to flow cytometry analysis with a FACS-Canto flow cytometer (BD Bioscience, USA).

### Immunofluorescence staining

The animals were anesthetized and transcardially perfused with 4% paraformaldehyde in phosphate buffer. Brains were removed and embedded in paraffin. Brain blocks were then sectioned into serial arrays of 3-μm-thick sections. The tissue sections were deparaffinized, rehydrated and subjected to antigen retrieval. Then, sections and cultured astrocytes were permeabilized with 0.4% Triton X-100 for 10 min and blocked with goat serum (cat. no. ab7481; Abcam, UK) for 1 h to eliminate nonspecific staining. Sections and cultured astrocytes were incubated with a mixture of a rabbit anti-TGF-β2 antibody (dilution 1:250, cat. no. ab113670; Abcam, UK) and rabbit anti-GFAP antibody (dilution 1:200, cat. no. ab7260; Abcam, UK) overnight at 4 °C. Alexa Fluor-conjugated secondary antibodies (cat. no. ab150077; Abcam, UK) were then incubated with the samples for 1 h at room temperature. The cell nucleus was stained with 0.1% Hoechst 33342 (Sigma–Aldrich, USA) for 5 min at room temperature. TGF-β2 and GFAP staining was observed using a Nikon Eclipse 80i microscope (Nikon Corporation).

### RNA sequencing, identification, and quantification of mouse circRNAs

Total RNA was extracted from six hippocampal tissues from each group. The RNA concentration and purity in each sample were quantified using a NanoDrop ND-1000 spectrophotometer (NanoDrop, Wilmington, DE, USA). RNA integrity was assessed using an Agilent 2100 instrument, with RIN > 7.0. Approximately 5 μg of total RNA was used to deplete ribosomal RNA according to the instructions of the Ribo-Zero™ rRNA Removal Kit (Illumina, San Diego, USA), followed by cDNA library construction. Next, deep sequencing was performed with an Illumina HiSeq 4000 instrument (LC Bio, China) according to the vendor's recommended protocol. First, Cutadapt was used to remove the reads that contained adaptor contamination, low-quality bases and undetermined bases. Then, sequence quality was verified using FastQC. We used Bowtie2 and Hisat2 to map reads to the genome of the species. The remaining reads (unmapped reads) were still mapped to the genome using TopHat fusion. CIRCExplorer2 and CIRI were first used for the de novo assembly of the mapped reads to circular RNAs; then, back splicing reads were identified in unmapped reads using TopHat fusion. All samples generated unique circular RNAs. The differentially expressed circRNAs were selected with log2 (fold change) > 1 or log2 (fold change) < − 1 and with statistical significance (*P* value < 0.05) using the R package edgeR.

### Cell transfection

For this experiment, miR-8114 mimic, miR-NC mimic, miR-8114 inhibitor, miR-NC inhibitor, specific small interfering RNAs (siRNAs) targeting circ_0001293 (si-Circ) and TGF-β2 (si-TGF-β2), and siRNA-negative control (si-NC) were purchased from Shanghai GenePharma (China). The sequences of circ_0001293 were inserted into a pcDNA3.1 plasmid to obtain the circ_0001293 overexpression plasmid pcDNA3.1-circ_0001293 (oe-circ), and an empty pcDNA3.1 plasmid was used as the negative control (oe-NC). Plasmid DNA, siRNA, miR-mimic or miR-inhibitor was transfected into astrocytes (1 × 10^5^), which were subcultured at a density of 80%, with Lipofectamine 2000 reagent (Invitrogen; Thermo Fisher Scientific, Inc., USA) at 37 °C. Forty-eight hours after transfection, the transfection efficiency was detected using RT‒qPCR and Western blotting, and then subsequent experiments were performed.

### Bioinformatics and luciferase reporter analyses

RNA22 (https://cm.jefferson.edu/rna22/Interactive/) online software was used to predict target binding sites of miRNAs in circRNAs and mRNAs. PmirGLO-circ_0001293-wild (circ-WT)/-mutant (circ-MUT) type and PmirGLO-TGF-β2-WT/-MUT type reporter plasmids were provided by Shanghai GenePharma Co., Ltd. HEK293 cells (2 × 10^5^/well) were cotransfected with the circ-WT/-MUT or TGF-β2-WT/-MUT plasmid and miR-NC mimic or miR-8114 mimic using Lipofectamine 2000 reagent (Invitrogen, USA) at 37 °C. At 48 h post-transfection, luciferase activity was determined using the dual-luciferase reporter assay system (Promega Corporation, USA). Firefly luciferase activities were normalized to *Renilla* luciferase activities.

### Enzyme-linked immunosorbent assay (ELISA)

The levels of the inflammatory cytokines TNF-α (Abcam, UK), IFN-γ (Abcam, UK), IL-6 (Abcam, UK), IL-1β (Abcam, UK), IL-10 (Abcam, UK), and TGF-β2 (Solarbio, China) in the hippocampus were measured using corresponding ELISA kits according to the manufacturer’s instructions. Absorbance was determined using a microplate spectrophotometer (BioTeke, China).

### RNA extraction and reverse transcription–quantitative PCR (RT‒qPCR)

Total RNA was extracted from cells using TRIzol® reagent (Invitrogen, USA) according to the manufacturer's protocol. First-strand cDNAs were synthesized from total RNA using the PrimeScript™ RT reagent kit (Takara Biotechnology Co., Ltd., Japan) according to the manufacturer’s instructions. RT–qPCR was subsequently performed using the SYBR-Green qPCR kit (Thermo Fisher Scientific, Inc., USA) according to the manufacturer’s protocols. The following primer sequences were used for RT‒qPCR: miR-8114, forward 5′-TCACCCATCTCCTCTCC-3′ and reverse 5′-TGTCGTGGAGTCGGC-3′; TGF-β2, forward 5′-CCAGGGGGAAGGAGGTCATA-3′ and reverse 5′-CTTCGGCAGACACGTGTTTG-3′; circ_0001293, forward 5′-AAATCCTCTGCAGCCCTTCC-3′ and reverse 5′-TTCATCTCTTGGTCGAGGCG-3′; U6, forward 5′-CTCGCTTCGGCAGCACA-3′ and reverse 5′-AACGCTTCACGAATTTGCGT-3′; and β-actin, forward 5′-CACTGTGCCCATCTACGAGG-3′ and reverse 5′-TAATGTCACGCACGATTTCC-3′. RT‒qPCR experiments were performed using an Applied Biosystems 7900HT Fast Real-time PCR system (Applied Biosystems; Thermo Fisher Scientific, Inc., USA). The relative expression levels were calculated using the 2-ΔΔCq method [35] and normalized to those of the internal reference genes β-actin (mRNA) and U6 (miRNA).

### Western blot

Tissues and cells were isolated, total proteins were extracted using RIPA buffer, and protein concentrations were measured using a BCA Protein Determination kit (Pierce Biotechnology, USA). Equal quantities of protein samples were separated on 10% SDS–PAGE gels and transferred to polyvinylidene difluoride membranes, which were then washed and blocked. Specific primary antibodies against TGF-β2 (1:1000; cat. no. ab205150; Abcam, UK), p-ERK (1:500; cat. no. ab47310; Abcam, UK), p-p38 (1:1000; cat. no. ab240335; Abcam, UK), p-JNK (1:1000; cat. no. ab124956; Abcam, UK), p–p65 (1:1000; cat. no. ab76302; Abcam, UK), and β-actin (1:5000; cat. no. ab8226; Abcam, UK) were incubated with membranes, followed by an incubation with an HRP-conjugated secondary antibody (1:5000; cat. no. ab20272; Abcam, UK). Antibody binding was detected using enhanced chemiluminescence reagent (Thermo Fisher Scientific, Inc., USA). ImageJ (Version 1.49; NIH, USA) was used to analyze the gray value of each band on the membrane.

### Cell viability

Forty-eight hours after cells were seeded in 96-well plates at a density of 2500 cells per well, cell viability was assessed using a CCK-8 assay (Solarbio, China) according to the manufacturer’s instructions. Absorbance was measured using a microplate spectrophotometer (BioTeke, China).

### Statistical analysis

All experiments were performed in triplicate. Statistical analyses were performed using GraphPad Prism 7 (GraphPad Software Inc., San Diego, CA, USA). The results are presented as the means ± standard deviations (SD). Two groups were compared with Student’s t test, and three or more treatments or groups were compared with one-way ANOVA followed by the Tukey–Kramer post hoc test. A P value < 0.05 was defined as statistically significant.

## Results

### HsTx2 suppresses PTZ-induced epilepsy in an animal model

**Fig. 1:**
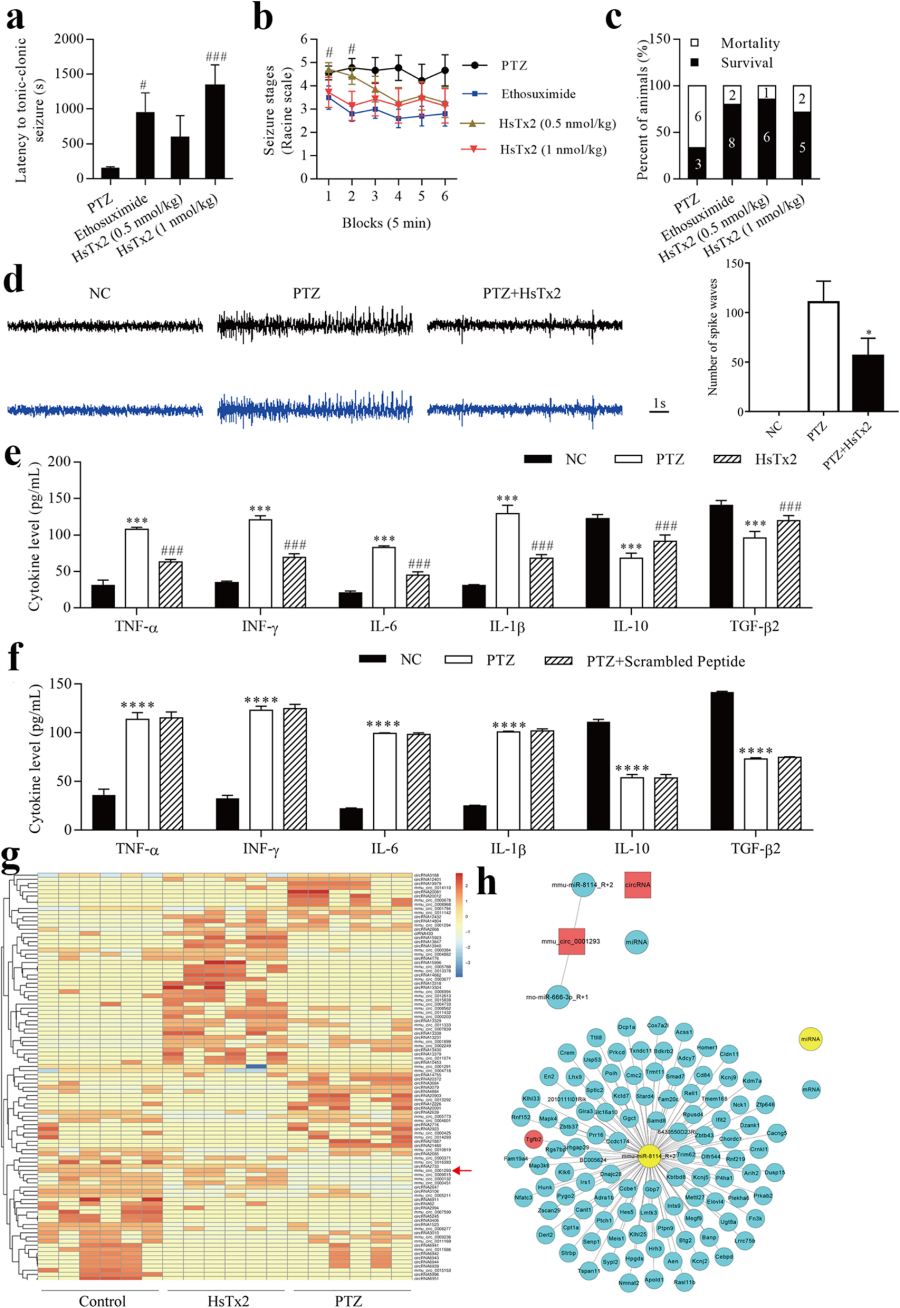
HsTx2 suppresses PTZ-induced epilepsy in an animal model. **a** Effect of HsTx2 on the latency to tonic‒clonic seizures in the PTZ group (*n* = 9), ethosuximide group (*n* = 10), 0.5 nmol/kg HsTx2 group (*n* = 7) and 1 nmol/kg HsTx2 group (*n* = 7). **b** Racine scores of the PTZ group (*n* = 9), ethosuximide group (*n* = 10), 0.5 nml/kg HsTx2 group (*n* = 7) and 1 nmol/kg HsTx2 group (*n* = 7). **c** Survival ratios in the PTZ group (*n* = 9), ethosuximide group (*n* = 10), 0.5 nmol/kg HsTx2 group (*n* = 7) and 1 nmol/kg HsTx2 group (*n* = 7). **d** Representative EEG recordings from the three groups of mice (*n* = 6) and histogram of the number of spike waves. **e** ELISAs were used to determine the levels of TNF-α, IFN-γ, IL-6, IL-10 and TGF-β2 after HsTx2 treatment in mouse brains (*n* = 6). **f** ELISAs were used to determine the levels of TNF-α, IFN-γ, IL-6, IL-10 and TGF-β2 after random scrambled peptide treatment in mouse brains (*n* = 3). **g** Heatmap of circRNAs (*n* = 6). **h** circ_0001293-related circRNA-miRNA‒mRNA negative correlation network. ^*^P < 0.05 compared with the PTZ group; ^***^P < 0.001 compared with the NC group; ^****^P < 0.0001 compared with the NC group; ^#^*P* < 0.05 and ^###^*P* < 0.001 compared with the PTZ group

As shown in our previous study, HsTx2 ameliorates cerebral ischemic injury in rats by exerting neuroprotective effects [15], suggesting that it may be a new potential ASD. The latency to tonic‒clonic seizures, seizure stage and survival of each group of mice are shown in Fig. 1a–c. Ethosuximide and HsTx2 increased the latency to tonic‒clonic seizures compared to the negative control, and 1 nmol/kg HsTx2 increased the latency to tonic‒clonic seizures compared to the positive control (Fig. 1a). Our spontaneous seizure analysis showed that both ethosuximide and HsTx2 decreased spontaneous seizure severity compared with the negative control, but 1 nmol/kg HsTx2 did not change spontaneous seizure severity compared with the positive control (Fig. 1b). The survival ratio was increased by both ethosuximide and HsTx2 (Fig. 1c). Compared with 0.5 nmol/kg HsTx2, greater antiseizure activity was observed for 1 nmol/kg HsTx2. Thus, 1 nmol/kg HsTx2 was used in subsequent assays. As shown in Fig. 1d, the amplitude of seizure spike waves was significantly increased in the PTZ group compared to the NC group. Interestingly, HsTx2 reduced the amplitude of seizure spike waves in PTZ-induced mice. HsTx2 decreased the levels of TNF-α, IFN-γ and IL-6 but increased the levels of IL-10 and TGF-β2 (Fig. 1e). A randomly scrambled peptide was used as a control. In Fig. 1f, the treatment of randomly scrambled peptide could not reverse the decrease of TNF-α, IFN-γ and IL-6, and the increased of IL-10 and TGF-β2 levels by PTZ stimulation. RNA-depleted total RNA sequencing was performed to profile circRNA expression and screen potential driver circRNAs involved in the effects of HsTx2 on epilepsy. The heatmap of identified differentially expressed circRNAs among hippocampal tissues from the control group, PTZ group and HsTx2 group is shown in Fig. 1g, and some of those differential circRNAs were validated to be related to the therapeutic effects of HsTx2 on epilepsy. We found that circ_0001293 expression was reduced by PTZ administration, but this change was reversed by HsTx2 treatment (Figs. 1g and 8a). Therefore, we further explored the underlying molecular mechanism responsible for the effects of circ_0001293 on astrocytes. CircRNAs have been shown to function as miRNA sponges in the cytoplasm [28]. We first predicted the potential target miRNAs in the CircNet database using bioinformatics to understand the actions of circ_0001293. As shown in Fig. 1h, circ_0001293 (red) was predicted to interact with mmu-miR-8114 and rno-miR-666-3p (blue), but rno-miR-666-3p is a circRNA in rats. Hence, miR-8114 was considered the miRNA target of circ_0001293. Subsequently, we predicted the potential target mRNAs (blue) of miR-8114 (yellow) to design the miRNA‒mRNA regulatory network using TargetScan and miRanda software. Interestingly, we identified TGF-β2 as a target gene of miR-8114. Ultimately, the circRNA–miRNA‒mRNA network of the circ_0001293/miR-8114/TGF-β2 axis was assessed in subsequent studies.

### circ_0001293 sponges miR-8114, and miR-8114 targets TGF-β2

**Fig. 2:**
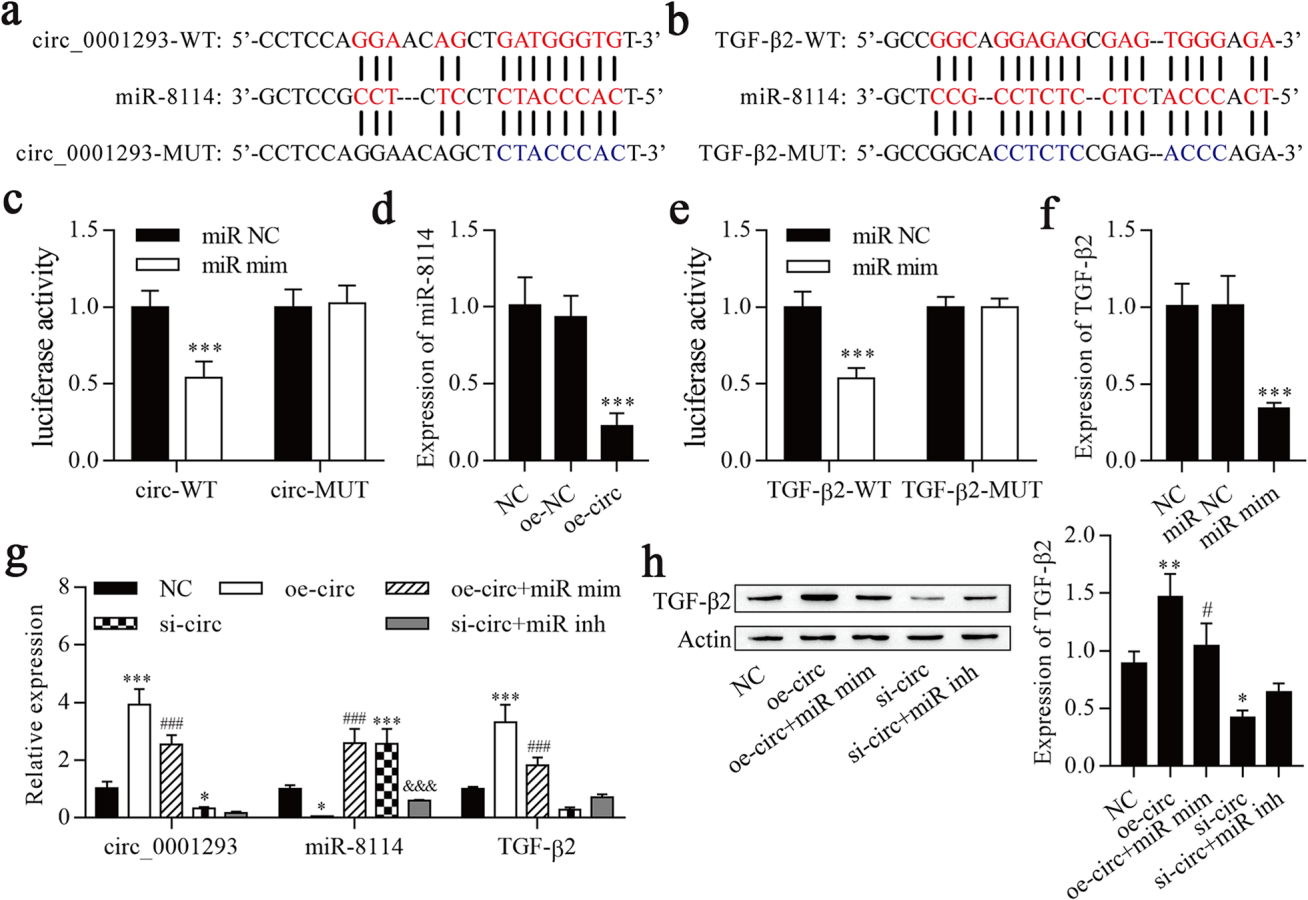
circ_0001293 sponges miR-8114, and miR-8114 targets TGF-β2. **a** Schematic representation of the miR-8114 binding site in circ_0001293-WT and circ_0001293-MUT. **b** Schematic representation of the miR-8114 binding site in TGF-β2-WT and TGF-β2-MUT. **c** Dual-luciferase reporter assays were performed and showed that miR-8114 targets circ_0001293. ^***^P < 0.001 compared with miR NC. **d** RT–qPCR was conducted to determine the expression of miR-8114. ^***^P < 0.001 compared with NC. **e** Dual-luciferase reporter assays were conducted and showed that miR-8114 targets TGF-β2. ^***^P < 0.001 compared with miR NC. **f** RT–qPCR was used to determine TGF-β2 expression. ^***^P < 0.001 compared with NC. **g** RT–qPCR was performed to determine the expression of circ_0001293, miR-8114 and TGF-β2. ^*^P < 0.05 and ^***^P < 0.001 compared with NC, ^###^*P* < 0.001 compared with oe-circ_0001293, and ^&&&^*P* < 0.001 compared with si-circ_0001293. **h** Levels of the TGF-β2 protein detected using Western blotting. ^**^P < 0.01 compared with NC; ^#^*P* < 0.05 compared with oe-circ_0001293. All values are presented as the means ± SD (*n* = 3)

Using the bioinformatics database starBase to search for potential targets, a putative interaction between miR-8114 and circ_0001293/TGF-β2 was identified, and the target binding sequence is shown in Fig. 2a and b. A dual-luciferase reporter assay revealed that circ-WT/TGF-β2-WT and miR-8114 mimic cotransfection significantly decreased the luciferase activities in HEK293 cells (Fig. 2c, e), while circ-MUT/TGF-β2-MUT and miR-8114 mimic cotransfection failed to alter the luciferase activity in HEK293 cells. Transfection of oe-circ into HEK293 cells significantly decreased miR-8114 expression (Fig. 2d), and transfection of miR-8114 mimic into HEK293 cells significantly decreased TGF-β2 expression (Fig. 2f). Subsequently, the circRNA–miRNA‒mRNA network of the circ_0001293/miR-8114/TGF-β2 axis was explored. RT–qPCR was conducted to examine the expression of circ_0001293, miR-8114 and TGF-β2, and the overexpression of circ_0001293 increased the expression of circ_0001293 and TGF-β2 and decreased miR-8114 expression, but these effects were reversed by the overexpression of miR-8114 (Fig. 2g). Knockdown of circ_0001293 reduced the expression of circ_0001293 and TGF-β2 and increased miR-8114 expression, but miR-8114 upregulation and TGF-β2 downregulation were reversed upon the downregulation of miR-8114. Western blot results showed increased and decreased levels of the TGF-β2 protein following circ_0001293 upregulation and downregulation, respectively, but these changes were reversed by miR-8114 mimic and miR-8114 inhibitor treatment, respectively (Fig. 2h). Based on these observations, circ_0001293 sponges miR-8114, and miR-8114 targets TGF-β2.

### HsTx2 alleviates IL-1β-induced astrocyte inflammation

**Fig. 3:**
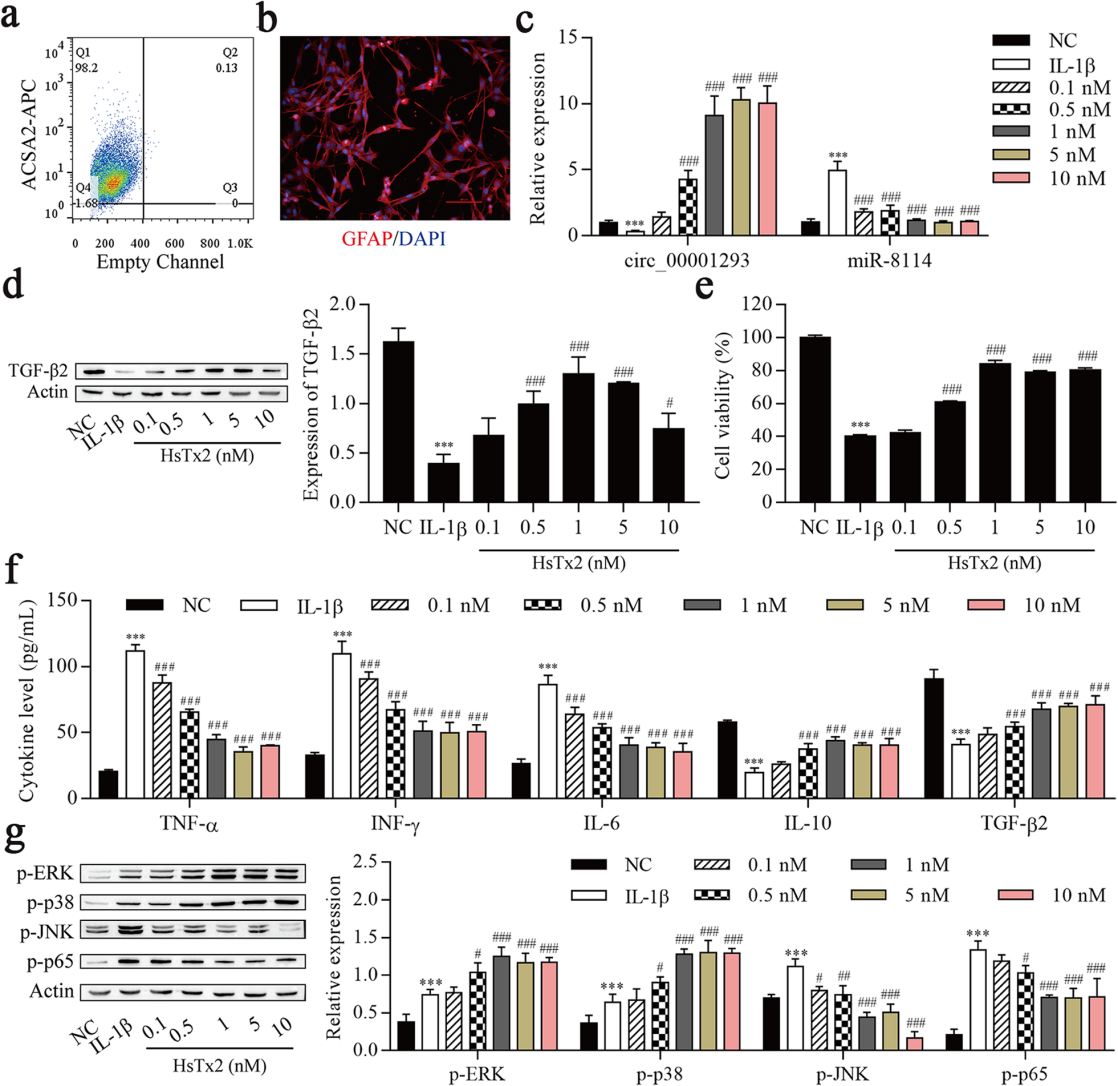
HsTx2 alleviates IL-1β-induced astrocyte inflammation. **a** Flow cytometry analysis of ACSA2-APC in ACSA2+ cells. **b** Immunofluorescence staining confirmed that purified adult astrocytes express GFAP. **c** Astrocytes were treated with different concentrations of HsTx2 (0.1, 0.5, 1, 5 or 10 nM) in the presence of IL-1β. RT–qPCR was used to determine the expression of circ_0001293 and miR-8114 (*n* = 3). HsTx2 increased the expression of circ_0001293 and reduced miR-8114 expression. **d** Astrocytes were treated with different concentrations of HsTx2 (0.1, 0.5, 1, 5 or 10 nM) in the presence of IL-1β. Levels of the TGF-β2 protein were examined using Western blotting (*n* = 3). HsTx2 increased the expression of TGF-β2. **e** Cell viability was detected using the CCK-8 assay (*n* = 3) with different concentrations of HsTx2 (0.1, 0.5, 1, 5 or 10 nM) in the presence of IL-1β. **f** ELISAs were used to determine the levels of TNF-α, IFN-γ, IL-6, IL-10 and TGF-β2 with different concentrations of HsTx2 (0.1, 0.5, 1, 5 or 10 nM) in the presence of IL-1β. **g** MAPK- and NF-κB-related proteins were detected using Western blotting (*n* = 3) with different concentrations of HsTx2 (0.1, 0.5, 1, 5 or 10 nM) in the presence of IL-1β. ^***^P < 0.001 compared with NC; ^#^*P* < 0.05, ^##^P < 0.01 and ^###^*P* < 0.001 compared with PTZ

Astrocytes were purified from hippocampal tissues to confirm the molecular mechanisms underlying the antiseizure effect of HsTx2. As shown in Fig. 3a, 98.2% of the microbead-labeled cells were ACSA2+. Immunofluorescence staining confirmed that over 90% of bead-bound cells expressed GFAP (Fig. 3b). These astrocytes were treated with different concentrations of HsTx2 (0.1, 0.5, 1, 5 or 10 nM) for 48 h to examine whether HsTx2 regulated the expression of circ_0001293, miR-8114 and TGF-β2 in IL-1β-stimulated astrocytes. As shown in Fig. 3c and d, HsTx2 increased the expression of circ_0001293 and TGF-β2 and reduced miR-8114 expression. Next, the viability and inflammation of astrocytes were determined. As shown in Fig. 3e, cell viability was reduced after IL-1β induction but increased by HsTx2 treatment. ELISA results showed that IL-1β not only increased the levels of TNF-α, IFN-γ and IL-6 but also decreased the levels of IL-10 and TGF-β2, which were reversed upon HsTx2 treatment (Fig. 3f). Various studies have shown that the role of nuclear factor kappa B (NF-κB) in different neurodegenerative diseases is related to inflammation [36]. Elevated levels of the transcription factor NF-κB in the epileptic brain induce the transcription of genes encoding proinflammatory mediators, such as IL-1β, IL-6 and TNF-α [37]. As shown in our previous study, HsTx2 also regulates the MAPK signaling pathway [15]. Thus, we investigated whether HsTx2 regulated the NF-κB and MAPK pathways in IL-1β-treated astrocytes. IL-1β increased the levels of p-ERK, p-p38, p-JNK and p-p65. The levels of p-ERK and p-p38 increased continuously after HsTx2 treatment, but the p-JNK and p-p65 levels were reduced (Fig. 3g). Compared with 0.1, 0.5, 5 and 10 nM HsTx2, the effects of 1 nM HsTx2 on TGF-β2 expression, cell viability, NF-κB pathway activation and inflammatory cytokine levels were significant. Thus, cells treated with 1 nM HsTx2 for 48 h were used in subsequent experiments.

### HsTx2 alleviates IL-1β-induced astrocyte inflammation by upregulating circ_0001293

**Fig. 4:**
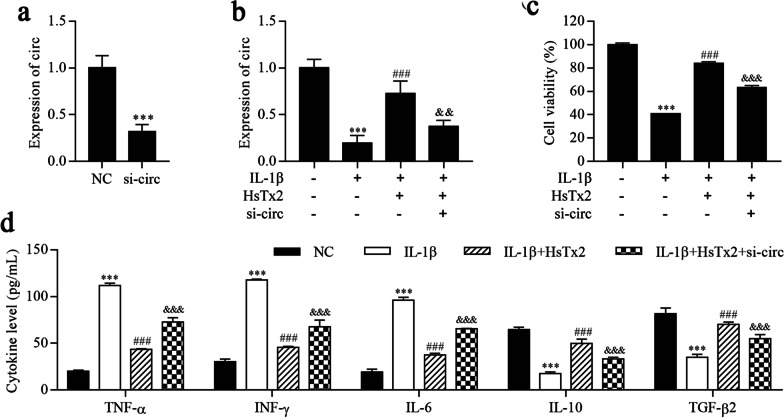
HsTx2 alleviates IL-1β-induced astrocyte inflammation by upregulating circ_0001293. **a**, **b** RT–qPCR was used to determine the expression of circ_0001293. **c** Cell viability was detected using the CCK-8 assay. **d** ELISAs were conducted to determine the levels of TNF-α, IFN-γ, IL-6, IL-10 and TGF-β2. ^***^P < 0.001 compared with NC; ^###^*P* < 0.001 compared with IL-1β; ^&&^*P* < 0.01 and ^&&&^*P* < 0.001 compared with IL-1β + HsTx2. All values are presented as the means ± SD (*n* = 3)

We constructed stable circ_0001293 knockdown cell lines using si-circ_0001293, and si-circ_0001293 significantly downregulated the expression of circ_0001293 (Fig. 4a). The results of the RT–qPCR analysis indicated that IL-1β reduced circ_0001293 levels, which was reversed by HsTx2 treatment, but circ_0001293 expression was finally repressed by si-circ_0001293 (Fig. 4b). Furthermore, cell viability and inflammatory cytokine levels were detected using the CCK-8 assay and ELISA, respectively. As shown in Fig. 4c, cell viability was reduced after IL-1β treatment but was alleviated by HsTx2 treatment, which was terminally inhibited by si-circ_0001293. The levels of the inflammatory cytokines TNF-α, IFN-γ and IL-6 were increased after IL-1β treatment but reduced by HsTx2 treatment, which was terminally increased by si-circ_0001293 (Fig. 4d). In contrast, IL-1β reduced the levels of IL-10 and TGF-β2, which were reversed by HsTx2 treatment, but the levels of IL-10 and TGF-β2 were ultimately repressed by si-circ_0001293. Based on these findings, HsTx2 alleviates IL-1β-induced astrocyte inflammation by upregulating circ_0001293.

### Overexpression of circ_0001293 alleviates IL-1β-induced astrocyte inflammation by downregulating miR-8114

**Fig. 5:**
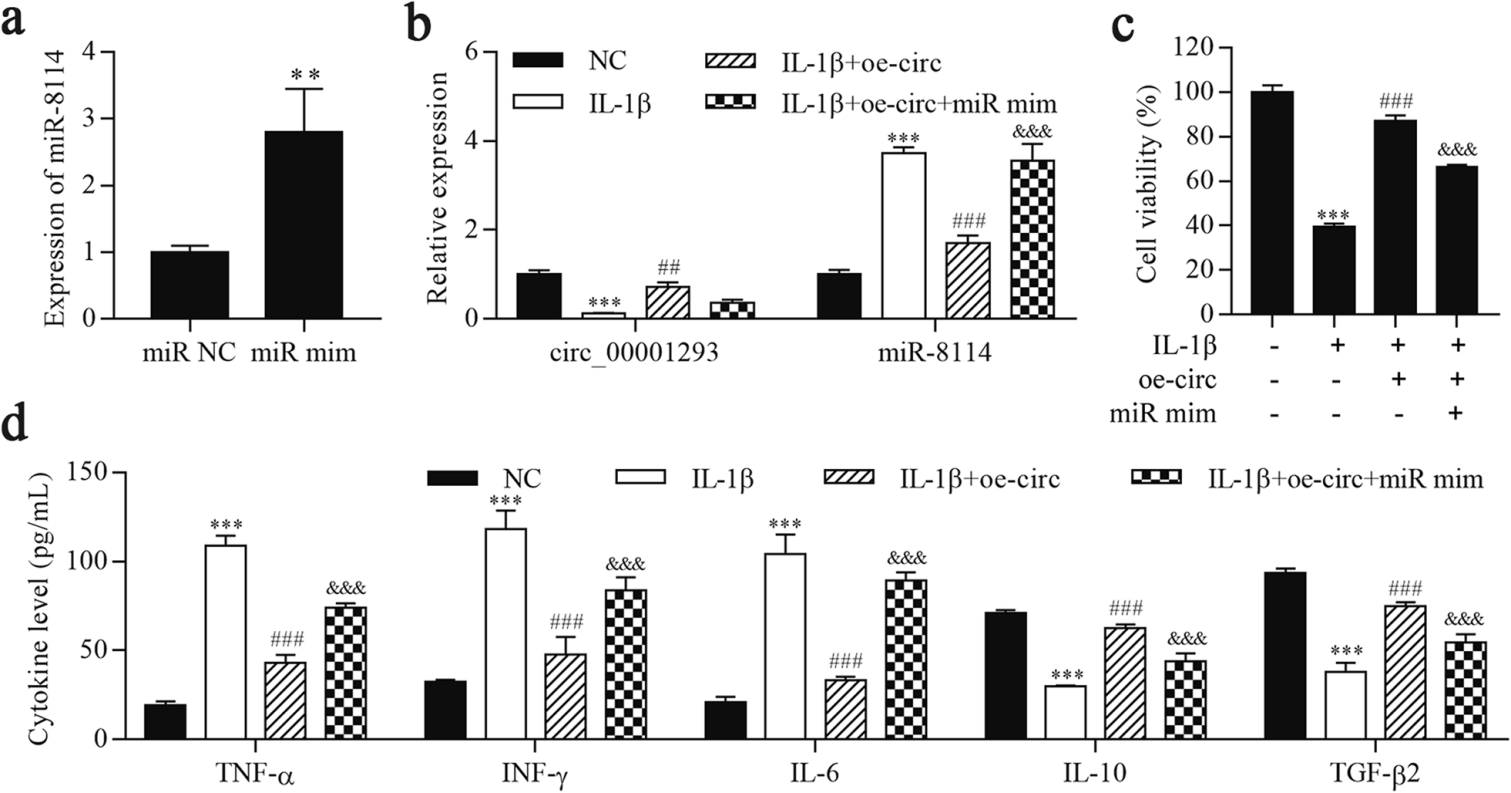
Overexpression of circ_0001293 alleviates IL-1β-induced astrocyte inflammation by downregulating miR-8114. **a**, **b** RT–qPCR was used to determine the expression of circ_0001293 and miR-8114. **c** Cell viability was detected using the CCK-8 assay. **d** ELISAs were conducted to determine the levels of TNF-α, IFN-γ, IL-6, IL-10 and TGF-β2. ^**^P < 0.01 and ^***^P < 0.001 compared with NC; ^##^*P* < 0.01 and ^###^*P* < 0.001 compared with IL-1β; ^&&&^*P* < 0.001 compared with IL-1β + oe-circ. All values are presented as the means ± SD (*n* = 3)

We next overexpressed miR-8114 in astrocytes and established cell lines stably overexpressing miR-8114 (Fig. 5a). The RT–qPCR results showed that circ_0001293 expression was significantly inhibited by IL-1β but increased after circ_0001293 overexpression, which was terminally repressed by miR-8114 mimic transfection (Fig. 5b). In contrast, miR-8114 expression was increased after IL-1β treatment but reduced by circ_0001293 overexpression, which was ultimately increased by the miR-8114 mimic. In addition, cell viability was reduced by IL-1β treatment but increased after circ_0001293 overexpression and was ultimately reduced by miR-8114 mimic transfection (Fig. 5c). Furthermore, ELISAs showed that the levels of the inflammatory cytokines TNF-α, IFN-γ and IL-6 were increased after IL-1β treatment but reduced upon the overexpression of circ_0001293 and increased after transfection of the miR-8114 mimic (Fig. 5d). In contrast, IL-1β reduced the levels of IL-10 and TGF-β2, changes that were reversed by oe_0001293, but the levels of IL-10 and TGF-β2 were reduced by miR-8114 overexpression. Therefore, circ_0001293 alleviates IL-1β-induced astrocyte inflammation by targeting miR-8114.

### Inhibition of miR-8114 alleviates IL-1β-induced astrocyte inflammation by targeting TGF-β2

**Fig. 6:**
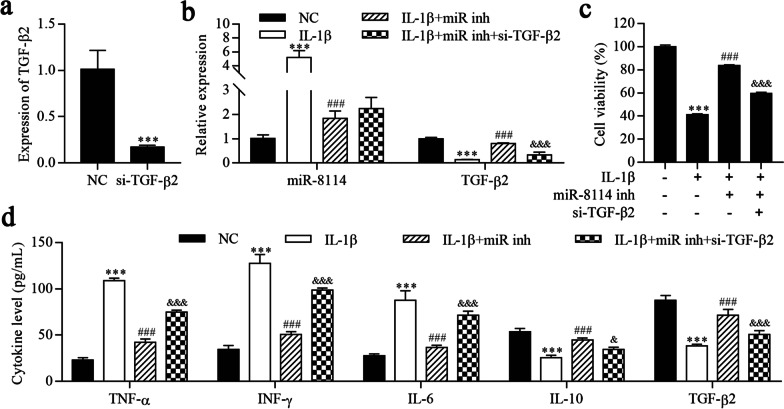
Inhibition of miR-8114 alleviates IL-1β-induced astrocyte inflammation by targeting TGF-β2. **a**, **b** RT–qPCR was used to determine the expression of miR-8114 and TGF-β2. **c** Cell viability was detected using the CCK-8 assay. **d** ELISAs were conducted to determine the levels of TNF-α, IFN-γ, IL-6, IL-10 and TGF-β2. ^***^P < 0.001 compared with NC; ^###^*P* < 0.001 compared with IL-1β; ^&&&^*P* < 0.001 compared with IL-1β + miR-8114 inhibitor. All values are presented as the means ± SD (*n* = 3)

We constructed stable TGF-β2 knockdown cell lines using si-TGF-β2, and si-TGF-β2 significantly downregulated TGF-β2 expression (Fig. 6a). As shown in Fig. 6b, miR-8114 expression was increased and TGF-β2 expression was reduced in the IL-1β group compared to the NC group. Inhibition of miR-8114 decreased miR-8114 expression and increased TGF-β2 expression, whereas TGF-β2 expression was reversed upon TGF-β2 knockdown. The CCK-8 results showed that IL-1β reduced cell viability, which was reversed upon miR-8114 inhibition. Cell viability was reduced by TGF-β2 knockdown (Fig. 6c). ELISAs showed that IL-1β increased the levels of TNF-α, IFN-γ and IL-6, but the levels of these cytokines were reduced by the miR-8114 inhibitor and ultimately increased by downregulating TGF-β2 (Fig. 6d). In contrast, IL-1β reduced the levels of IL-10 and TGF-β2, changes that were reversed by the miR-8114 inhibitor, but the levels of IL-10 and TGF-β2 were finally reduced by downregulating TGF-β2. Collectively, these results suggest that miR-8114 inhibition alleviates IL-1β-induced astrocyte inflammation by targeting TGF-β2.

### HsTx2 alleviates IL-1β-induced astrocyte inflammation via the circ_0001293/miR-8114/TGF-β2 axis

**Fig. 7:**
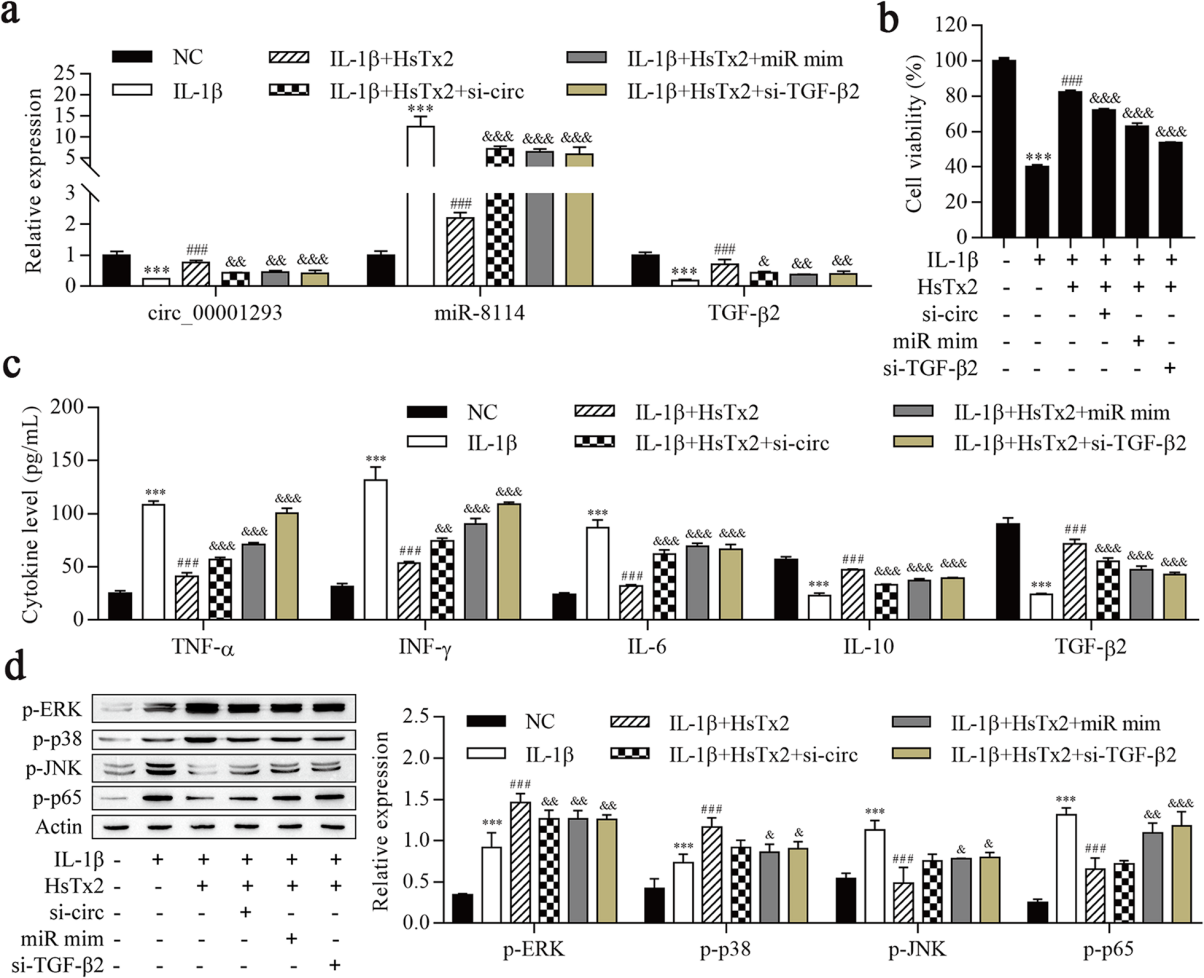
HsTx2 alleviates IL-1β-induced astrocyte inflammation via the circ_0001293/miR-8114/TGF-β2 axis. **a** RT–qPCR was used to determine the expression of circ_0001293, miR-8114 and TGF-β2. **b** Cell viability was detected using the CCK-8 assay. **c** ELISAs were conducted to determine the levels of TNF-α, IFN-γ, IL-6, IL-10 and TGF-β2. **d** Levels of MAPK- and NF-κB-related proteins were determined using Western blotting. ^***^P < 0.001 compared with NC; ^###^*P* < 0.001 compared with IL-1β; ^&^*P* < 0.05, ^&&^*P* < 0.01 and ^&&&^*P* < 0.001 compared with IL-1β + HsTx2. All values are presented as the means ± SD (*n* = 3)

We next examined the expression of circ_0001293 and its downstream genes miR-8114 and TGF-β2 to further validate whether HsTx2 exerts its function via the circ_0001293/miR-8114/TGF-β2 axis. As shown in Fig. 7a and b, IL-1β reduced the expression of circ_0001293 and TGF-β2, and the cell viability. HsTx2 treatment reversed these changes, and the expression of circ_0001293 and TGF-β2 was repressed in cells transfected with si-circ_0001293, miR-8114 mimic and si-TGF-β2. In contrast, miR-8114 expression was increased after IL-1β treatment but reduced by HsTx2 treatment, and its expression was increased in cells transfected with si-circ_0001293, miR-8114 mimic and si-TGF-β2. ELISA results showed that IL-1β not only increased the levels of TNF-α, IFN-γ and IL-6 but also decreased the levels of IL-10 and TGF-β2. HsTx2 treatment reduced the increases in TNF-α, IFN-γ and IL-6 levels and reversed the decrease in IL-10 and TGF-β2 levels after IL-1β stimulation, and the effects of HsTx2 were reversed after the transfection of si-circ_0001293, miR-8114 mimic and si-TGF-β2 (Fig. 7c). Western blot results showed that IL-1β induced increased p-ERK and p-p38 levels, and the increases were continuous with HsTx2 treatment, but si-circ_00001293, miR-8114 mimic and si-TGF-β2 reversed the effects of HsTx2 on p-ERK and p-p38 levels (Fig. 7d). IL-1β increased the levels of p-JNK and p-p65, HsTx2 treatment reversed these changes, and si-circ_00001293, miR-8114 mimic and si-TGF-β2 transfection ultimately increased the levels of these proteins. These data suggest that HsTx2 alleviates IL-1β-induced astrocyte inflammation via the circ_0001293/miR-8114/TGF-β2 axis.

### HsTx2 alleviates the progression of epilepsy via the circ_0001293/miR-8114/TGF-β2 axis

**Fig. 8:**
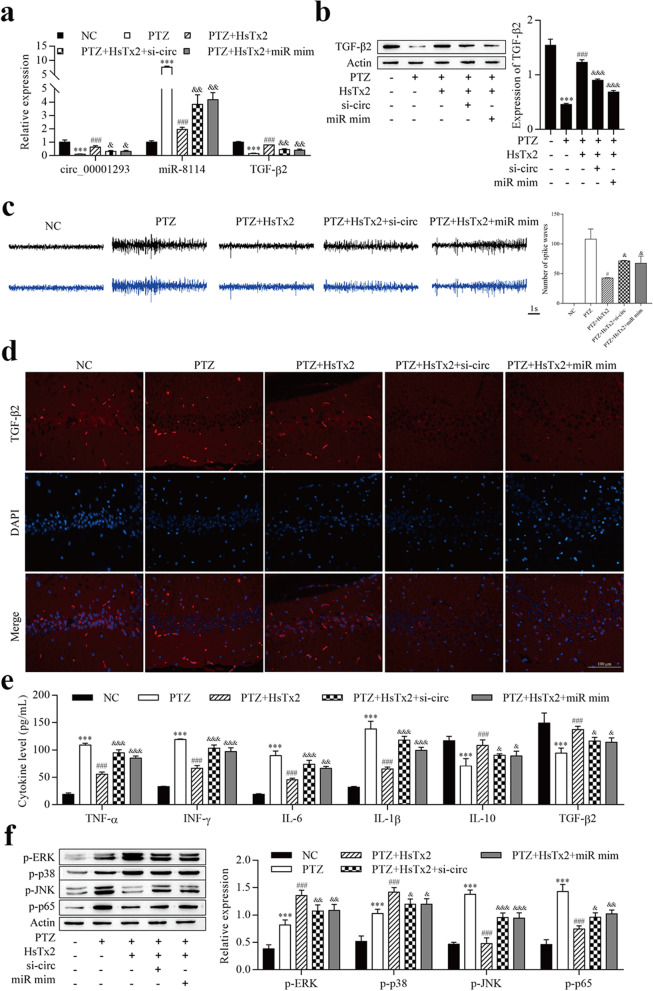
HsTx2 alleviates the progression of epilepsy via the circ_0001293/miR-8114/TGF-β2 axis. **a** RT–qPCR was used to determine the expression of circ_0001293, miR-8114 and TGF-β2. **b** Levels of the TGF-β2 protein were examined using Western blotting. **c** Representative EEG recordings from the five groups of mice and histogram of the number of spike waves. **d** Levels of the TGF-β2 protein were examined using immunofluorescence staining. **e** ELISAs were used to determine the levels of TNF-α, IFN-γ, IL-6, IL-10 and TGF-β2. **f** Levels of MAPK- and NF-κB-related proteins were determined using Western blotting. ^***^P < 0.001 compared with the NC group; ^#^*P* < 0.05 and ^###^*P* < 0.001 compared with the PTZ group; ^&^*P* < 0.05, ^&&^*P* < 0.01 and ^&&&^*P* < 0.001 compared with the PTZ + HsTx2 group. All values are presented as the means ± SD (*n* = 6)

We next assessed and confirmed whether the potential antiseizure activity of HsTx2 was mediated by the circ_0001293/miR-8114/TGF-β2 axis in vivo. Epileptic mice were injected with HsTx2, lenti-si-circ_0001293 or miR-8114 mimic, and the expression levels of these genes were analyzed. As shown in Fig. 8a, the expression of circ_0001293 and TGF-β2 was inhibited by PTZ but increased after HsTx2 treatment; their expression was repressed by si-circ_0001293 and miR-8114 mimic. In contrast, miR-8114 expression was increased after PTZ treatment but reduced by HsTx2 treatment, and its expression was increased by si-circ_0001293 and miR-8114 mimic. Both immunofluorescence staining and Western blotting revealed reduced levels of the TGF-β2 protein in animals receiving PTZ, HsTx2 treatment increased its levels, and si-circ_0001293 and miR-8114 mimic decreased levels of the TGF-β2 protein (Fig. 8b and d). In addition, scalp EEG results showed a significant decrease in the amplitude of seizure spike waves in the HsTx2 group compared to the PTZ group, but these effects of HsTx2 were reversed by si-circ_0001293 and miR-8114 mimic (Fig. 8c). ELISA results showed that PTZ increased the levels of TNF-α, IFN-γ, IL-6 and IL-1β, which were reversed by HsTx2 treatment, but the levels of TNF-α, IFN-γ, IL-6 and IL-1β were increased by si-circ_0001293 and miR-8114 mimic (Fig. 8e). In contrast, PTZ reduced the levels of IL-10 and TGF-β2, HsTx2 treatment reversed these changes, and the levels of IL-10 and TGF-β2 were reduced by si-circ_0001293 and miR-8114 mimic. In addition, Western blot results showed that PTZ induced increased p-ERK and p-p38 levels, and the increase was continuous with HsTx2 treatment, but si-circ_00001293 and miR-8114 mimic reversed the effects of HsTx2 on p-ERK and p–p38 levels (Fig. 8f). PTZ increased the levels of p-JNK and p–p65, HsTx2 treatment reversed these changes, and the levels of these proteins were increased by si-circ_00001293 and miR-8114 mimic. Taken together, HsTx2 alleviates epilepsy-induced inflammation via the circ_0001293/miR-8114/TGF-β2 axis.

## Discussion

A large number of bioactive peptides have been identified in recent years [38–43]. Peptides are also an important component of toxins and venoms produced by different organisms. Several toxin-derived peptides have been developed as drugs for the management of diabetes, hypertension, chronic pain, and other medical conditions [44]. Scorpion toxins have been used as exogenous molecular probes to analyze the endogenous molecular mechanism [45]. For example, a toad toxin-resistant snake (*Thamnophis elegans*) expresses high levels of mutant Na(^+^)/K(^+^)-ATPase mRNA in cardiac muscle [46]. Scorpion peptides have been reported to exhibit antiseizure activity in an epileptic model [47–49]. Based on our results, HsTx2 suppresses the progression of PTZ-induced epilepsy in a mouse model and alleviates IL-1β-induced astrocyte inflammation. Our results indicate that HsTx2 is a peptide drug with antiseizure activity. However, the molecular mechanism underlying the antiseizure effect of HsTx2 remains largely unknown. We identified circRNA expression profiles in HsTx2-treated tissues using RNA-seq. Among these differentially expressed circRNAs in the epilepsy model, we defined a critical role for a circRNA, circ_0001293, in HsTx2-mediated alleviation of the progression of epilepsy. Mechanistically, circ_0001293 sponged miR-8114 and upregulated TGF-β2 expression. Thus, our findings revealed that HsTx2 attenuates the progression of epilepsy by regulating the circ_0001293/miR-8114/TGF-β2 axis.

Notably, circRNAs have been reported to play numerous roles in gene regulation and other cellular processes [28, 50]. Importantly, circRNAs exert their functions via diverse mechanisms, including serving as miRNA or protein inhibitors ('sponges'), regulating protein function or being translated themselves. CircRNAs have been reported to be important endogenous genes that regulate epilepsy progression [29–31]. For example, circ_ANKMY2, circ_DROSHA and circ_0003170 regulate epilepsy progression [30, 31, 51]. Based on our knowledge, although circ_0001293 has not been identified in humans at present, in view of the relationship between circ_0001293 and seizures in the PTZ-induced mouse model, there is a reason to believe that the in-depth exploration of circ_0001293 may help us to further understand seizures and new mechanisms and may also provide potential targets for the treatment of seizures.

Previous studies have documented changes in miRNA levels in the hippocampus of patients with temporal lobe epilepsy and neural tissues from animal models of epilepsy [52], such as miR-106b-5p, miR-421 and miR-181b [30, 31, 51, 53]. In the present study, miR-8114 was identified as a circ_0001293-binding miRNA. Dysregulation of miR-8114 facilitates the epigenetic regulation of AQP2 and results in nephrogenic diabetes insipidus [54]. Here, miR-8114 expression was increased after IL-1β- and PTZ-induced epilepsy but reduced by HsTx2 treatment. Interestingly, overexpression of miR-8114 reversed the effects of HsTx2, and oe-circ_0001293 attenuated the progression of epilepsy.

Binding of miRNAs to the 3′ UTR of mRNAs inhibits gene expression post-transcriptionally [55]. We aimed to better identify the downstream target genes regulated by miR-8114 in the hippocampus of epilepsy. We finally found that TGF-β2 was the target of miR-8114 using the online software starBase. In humans, the TGF-β superfamily comprises 33 members that are subclassified into several subfamilies [56]. We observed that IL-1β reduced TGF-β2 levels in astrocytes, HsTx2 treatment reversed this change, and TGF-β2 levels were increased by si-circ_0001293, miR-8114 mimic and si-TGF-β2, suggesting that HsTx2 reduced astrocyte release of TGF-β2 by regulating the circ_0001293/miR-8114/TGF-β2 axis. The MAPK and NF-κB pathways are activated in subjects with epilepsy [57–59]. HsTx2 further increased the levels of p-ERK and p-p38 but reduced the levels of p-JNK and p-p65, suggesting that HsTx2 activates the MAPK signaling pathway and inhibits the NF-κB signaling pathway. We observed a similar trend in IL-1β-treated astrocytes. Previous studies have shown that TGF-β2 regulates the MAPK and NF-κB pathways [15, 60, 61]. Taken together, TGF-β2 is a novel target of HsTx2 mediating its antiseizure activity.

The effect of HsTx2 on ion channels was tested in previous experiments. Because direct effects of HsTx2 on ion channels were not found (data not shown), it is likely that anti-inflammatory effects are involved. Inflammatory responses are important in the development of epilepsy. Inflammatory cytokines are present in small amounts in the brain, but their levels increase after seizures [62, 63]. The dysregulation of glial immune inflammation is a common factor contributing to seizures. Meanwhile, acute seizures upregulate the production of proinflammatory cytokines in microglia and astrocytes, triggering a cascade of downstream inflammatory mediators [64]. IL-1β can be detected in microglia after seizures, but its expression disappears after several hours [65]. Microglia are activated in human epilepsy, including lamouson encephalitis. Notably, the degree of microglial activation is correlated with the seizure frequency and disease duration of drug-resistant epilepsy. Microglia and astrocytes may also remain activated morphologically after inhibiting cytokine synthesis in experimental epileptic tissues [66]. Due to the limitations of time and experimental cost, astrocytes were selected for research in this study. We would like to explore the inflammatory response of the microglial system in future studies. Astrocytes play various roles in the development and resolution of neuroinflammation through numerous mechanisms [67]. Reactive astrocytes exhibit both proinflammatory neurotoxic and anti-inflammatory neuroprotective phenotypes [68–70]. TNF-α, IFN-γ and IL-6 have been shown to activate proinflammatory astrocytes and cause a secondary inflammatory response [69]. Anti-inflammatory cytokines such as IL-4, IL-13, and IL-10 may activate the neuroprotective functions of astrocytes, and these activated astrocytes may release IL-4, IL-10, and TGF-β [71]. In the present study, TNF-α, IFN-γ and IL-6 levels increased in the epilepsy models, while IL-10 and TGF-β2 levels decreased, and these effects were reversed by HsTx2 treatment. In contrast, the random scrambled peptide did not regulate inflammatory factors in seizure models. TGF-β2 is a member of the TGF-β family, and TGF-β2 signaling regulates the secretion of proinflammatory cytokines [72]. In this study, we observed decreased levels of the TGF-β2 protein in epilepsy models, but these changes were reversed by HsTx2 treatment. Therefore, HsTx2 induces the neuroprotective activation of astrocytes.

## Conclusions

In summary, HsTx2 treatment significantly protects against the progression of epilepsy through a mechanism that mainly depends on anti-inflammatory activity by regulating the circ_0001293/miR-8114/TGF-β2 axis. These findings suggest that HsTx2 is an antiseizure peptide that should be further investigated for use in the treatment of epilepsy.

## Data Availability

The data sets used and/or analyzed during the current study are available from the corresponding author on reasonable request.

## Abbreviations

ASDs: Available antiseizure drugs
PTZ: Pentylenetetrazol
TGF-β2: Transforming growth factor beta 2
IL-Iβ: Interleukin-1β
TNF-α: Tumor necrosis factor-α
CNS: Central nervous system
circRNAs: Circular RNAs
EEG: Electrophysiological
siRNAs: Small interfering RNAs
SD: Standard deviations
